# Supplementary material for: Numb Chin Syndrome in Sickle Cell Disease: A Systematic Review and Recommendations for Investigation and Management
Source: Diagnostics (Basel). 2022 Nov 24;12(12):2933. doi: 10.3390/diagnostics12122933 (PMC9776680; doi:10.3390/diagnostics12122933)
Supplement: Supplementary file 1 [file diagnostics-12-02933-s001.zip › diagnostics-2002312-supplementary.pdf]

Supplementary Table S1. Publications identified by the systematic review of numb chin syndrome in sickle cell disease. Individual patient characteristics, clinical presentation, management, and disease evolution are reported, when available.

Author/Year	Country	Age (yrs)	Sex	SCD genotype	Clinical Presentation	Relevant workup	Hct (%)	Duration of symptoms (months)	Treatments
Konotey-Ahulu, 1972	Ghana	23	F	SC	Unilateral (4 patients) or bilateral (1 patient) burning sensation of the lower lip followed by numbness in the mental nerve distribution after VOC involving the mandibular region	-	-	18	-
		37	F	SC					
		23	M	SC					
		31	F	SS					
		33	M	SS					
Kirson, 1979	United States	18	M	SC	Pain crisis involving the mandibular region, with concomitant unilateral numbness in the mental nerve distribution	Mandibular X-ray: questionable radiolucency in the superior portion of the left ramus, extending into the subcondylar region.<br><br>Biopsy: no signs of malignancy	38.6	9	Standard treatment of VOC
		29	M	S/β-thalassemia	Generalized pain crisis, involving the mandibular region, followed by numbness in the mental nerve distribution	Mandibular X-ray: no abnormality	37.8	1	Standard treatment of VOC
Friedlander, 1980	France	40	M	SS	Generalized pain crisis, involving the mandibular region, followed by numbness in the mental nerve distribution	Mandibular X-ray: 2 by 1 cm ovoid radiolucency consistent with acute bony infarct	29	>12	Standard treatment of VOC
Seeler, 1982	United States	11	F	-	Acute pain crisis not involving the mandible, followed by bilateral swelling over the rami of the mandible, followed by anesthesia to the left chin and lip	Mandibular X-ray: no abnormality<br><br>Viral titers: negative	-	4	Standard treatment of VOC
Patton, 1990	United States	34	M	-	Pain in the left jaw followed by paresthesia in the distribution of the left inferior alveolar and mental nerves in the context of mandibular osteomyelitis and bone necrosis	Mandibular X-ray: diffuse lytic changes with indistinct, fuzzy trabeculae, and development of radiolucent area and mottling consistent with subacute osteomyelitis.<br><br>Pathology: extensive subacute osteomyelitis with necrosis, dystrophic calcification, and degenerative changes in an entrapped myelinated nerve	27.5	-	Standard treatment of VOC<br><br>Tooth extraction, debridement of soft tissue mass<br><br>Treatment for infection
Gregory, 1994	England	27	M	SS	2 months after molar extraction, and 1 month after a severe VOC, patient developed paresthesia of the lower lip	Mandibular X-ray: step ladder trabeculation of alveolar bone consistent with sickle disease<br><br>Head CT-scan: no evidence of alternative explanation for mandibular nerve neuropathy, loss of normal cortical	-	>24	Standard treatment of VOC

						condensation around the right mental canal, although it was present on the left side<br><br>Radionuclide bone scan: slightly increased tracer uptake in the right mandibular molar region indicative of infarction			
Stevenson, 2004	England	33	F	-	Post-partum pain crisis not involving the mandibular region, accompanied by paresthesia and anesthesia of both sides of the lower jaw	Mandibular X-ray: no abnormalities<br><br>Cerebral MRI: no evidence of a space-occupying lesion or abnormality along the intra-cranial or extra-cranial course of the trigeminal nerve<br><br>Radionuclide bone scan: increased tracer uptake in the skull and the periarticular regions of the long bones, possibly due to reactive marrow hyperplasia, secondary to sickle cell disease	-	>36	Standard treatment of VOC
Mestoudjian, 2008	France	25	M	-	Pain crisis with pain affecting the face, associated with numbness of the left lip		-	>1	Standard treatment of VOC
		19	M	SC	Limb pain in the context of severe sickle crisis and multiorgan failure, followed by numbness of the left lip	Cerebral MRI: no abnormality	-	12	Exchange Transfusion
Robbins, 2009	United States	15	M	-	Acute painful neuropathy in the right mental nerve distribution followed by chin hypoesthesia in the context of sickle cell crisis	Cerebral MRI: no abnormality	-	>0.5	Standard treatment of VOC<br><br>Pregabalin
Hamdoun, 2012	United States	15	M	-	Lumbar pain crisis in the context of pneumonia/acute chest syndrome followed by headache and later numbness of bilateral chin and lip.	Head CT-scan: normal except for moderate paranasal sinus disease<br><br>MRI: Increased T2 signal in both mandibular rami with small subperiosteal fluid collection abutting the medial aspects of the rami. No enhancement on T1 images with contrast.<br><br>Bone scan: no abnormality in the mandible area	22.5	2	Treatment for infection and acute chest syndrome
Erdogan, 2013	Turkey	23	M	-	Generalized pain crisis including the mandible accompanied by bilateral lower lip and chin numbness	CT-scan: no abnormality	26.9	6	Transfusion
Bariha, 2018	India	18	M	-	Acute pain crisis in hips and knee followed by numbness of chin and lower lip and decreased sensation of touch in lower incisors and canine teeth	Mandibular X-ray: no abnormality<br><br>Head CT-scan: no abnormality<br><br>Cerebral MRI: no abnormality	-	-	-

		38	M	-	Numbness of chin, lower lip, and gingival mucosa in between two lower canine teeth	Head CT-scan: no abnormality<br><br>Cerebral MRI: no abnormality	-	-	-
Ramsay, 2021	Jamaica	17	F	SS	Acute pain crisis with vomiting and diarrhea, followed by jaw pain with numbness of cheeks. The pain improved but numbness persisted with mild swelling of the left mandible.	-	-	3	Standard treatment of VOC
		29	F	SC	Acute pain crisis including the mandible, in context of pneumonia/acute chest syndrome, followed by numbness of the left lip and chin.	Mandibular X-ray: no abnormality<br><br>Head CT-scan: no abnormality<br><br>Lumbar puncture: no abnormality	-	>132	-
		48	M	SS	Acute pain crisis including the mandible, with purulent discharge of the inner aspect of the right cheek followed by numbness of the right lip, chin, and jaw.	Head CT-scan: no abnormality	-	>168	No Treatment
		28	F	SS	Acute pain crisis including the mandible, followed by numbness to the lower lip and chin	-	-	0.07	Standard treatment of VOC
		33	F	SS	Two separate episodes in the first and second trimesters of her pregnancy: 1st occurred with sudden onset of numbness starting at the right jaw and moving across both sides of the lips, chin, mouth, and lower face, with swelling of the lips.<br>2nd episode occurred while she was admitted for a vaso-occlusive crisis including the mandible.	-	-	0.03	Standard treatment of VOC
		25	F	S/β-thalassemia	Severe tooth pain in context of dental filling damage, followed by swelling and numbness to both sides of the lips, jaw, cheeks, and inside of mouth.	-	-	>4	Standard treatment of VOC
		19	M	SS	Vaso-occlusive pain crisis including the mandible, with facial swellings followed by multiple oral cavity abscesses 6 months later.	-	-	8	-
		19	F	SS	Numbness in the lips and chin along with generalized pain including the mandible.	-	-	-	Standard treatment of VOC
		20	F	SS	Right-sided jaw swelling and numbness along with a vaso-occlusive crisis including the mandible.	-	-	-	Standard treatment of VOC
		59	F	SS	Numbness to the chin and generalized pain including the mandible.	-	-	-	-
		17	F	S/β-thalassemia	Severe headaches and pain to the face and upper limbs followed by swelling to the face and lips. Reported tenderness over the lower molars and hypoesthesia over the chin after pain resolution.	-	-	-	-
		35	M	SS	Painful vaso-occlusive crisis involving the mandible and numbness to the lower lip and chin.	Patient reported an MRI scan of the brain done which showed a “right-sided brain lesion” was done 2 years earlier	-	-	Standard treatment of VOC

		60	F	SC	Type II diabetic patient with a history of breast and liver cancer presents with chin numbness (decreased sensation to the left side of the chin), shortness of breath and productive cough.	-	-	>2	-
Cherry-Peppers, 1992	United States	34	F	-	Pain in left jaw and pain in the lower back and legs that resolved after transfusion.	Pulp Test: Non-vitality of teeth 19,20,21,22, and 30<br><br>Mandibular X-ray: Marked thinning of cortices in the area apical to #18 and in the right mandible inferior to #28 extending to #31. Radiolucent area apical to #18. Mass apical to #18 around the roots. 3cm lesion (diameter). Small occlusal amalgam on tooth #18.	18.9	>18	Standard treatment of VOC

Abbreviations: - = information was not reported, MRI = magnetic resonance imaging, VOC = venoocclusive crisis.
